# Case study: Monitoring the quality of cataract surgery at the Presbyterian Eye Clinic Acha-Bafoussam

**Published:** 2014

The Presbyterian Eye Clinic Acha-Bafoussam is one of the leading hospitals in Central Africa for high-volume cataract surgery. However, until 2011, there was no monitoring of the quality of cataract surgery and we did not know how we compared against the WHO standards.

Monitoring of outcomes of cataract surgery was introduced to the hospital with support from the London School of Hygiene and Tropical Medicine. It was a challenge to the entire eye care team as this was the first time they had experienced audit.

Data collected during ongoing audits highlights areas for improvement. The information collected is reviewed periodically and discussed with relevant members of the team. Appropriate actions are then taken to address areas of concern. Over the last four years there has been a steady improvement in clinical outcomes, patient safety and patient experience.

We have focused on improving VA testing, biometry and the supply of consumables (including intraocular lenses [IOLs] in a variety of dioptric powers), ensuring continuous curvilinear capsulorrhexis for most cases, introducing phacoemulsification, and improving counselling, ophthalmic theatre procedures and case selection (so that complicated cases are operated by the more experienced surgeons).

**Faustin Ngounou**, Medical Director

